# Effect of a probiotic supplement (*Bacillus subtilis*) on struggling behavior, immune response, and meat quality of shackled broiler chickens exposed to preslaughter stress

**DOI:** 10.1016/j.psj.2024.104051

**Published:** 2024-06-28

**Authors:** A.A. Mohammed, M.A. Mahmoud, R.S. Zaki, H.W. Cheng

**Affiliations:** ⁎ Department of Behavior and Management of Animals, Poultry and Aquatics, Faculty of Veterinary Medicine, Assiut University, Assiut 71526, Egypt; † Department of Animal Hygiene, Faculty of Veterinary Medicine, Assiut University, Assiut 71526, Egypt; ‡ Department of Meat Hygiene, Faculty of Veterinary Medicine, New Valley University, New Valley 72711, Egypt; § USDA Agricultural Research Service, West Lafayette, IN 47907, USA; # Department of Animal Husbandry and Livestock Development, School of Veterinary Medicine, Badr University in Assiut, Assiut, Egypt

**Keywords:** probiotic, preslaughter stress, welfare, immunity, meat quality, broiler chicken

## Abstract

This study aimed to investigate the impact of a dietary probiotic supplement on struggling behavior, immune response, and meat quality of shackled broiler chickens exposed to preslaughter stress. Two hundred and ten 1-day-old male Ross 708 broiler chicks were divided among 21 floor pens (10 chicks per pen). The pens were randomly distributed to 1 of 3 dietary treatments containing a probiotic, *Bacillus subtilis****,*** at 0 (control), 0.25 (0.25×), and 0.5 (0.5×) g/kg (n = 7). At the end of the experiment (d 35), birds were transported for a journey of 80 km to the abattoir, each crate contained 5 pen mates, 2 birds of them (2 bird per crate, total 14 birds per treatment) were randomly selected for testing. Struggling behavior measurements began after the birds had arrived at the abattoir. Serum and muscle samples (right leg and breast) were collected for immune response and meat quality parameters. The results indicated that probiotic supplemented broilers had lower breast muscle protein carbonyls and serum levels of IgM but higher breast muscle total antioxidant capacity (**TAC**) compared to those of controls. In addition, probiotic supplemented broilers’ leg and breast muscle had higher color lightness and greater water holding capacity (**WHC**%) with lower cooking loss (**CL**) and lower pH values (*P* < 0.05). Probiotic supplemented broilers’ breast and leg meat was also tastier (*P* < 0.05) compared to controls. There were no treatment effects on other measured parameters including struggling behavior, serum IgA and IgG concentrations, and breast muscle malondialdehyde (**MDA**) (*P* > 0.05). These results suggest that the probiotic supplement could be an alternative management tool for promoting broiler health and welfare by modifying immune response and meat quality.

## INTRODUCTION

Struggle (primarily wing flapping) of shackled birds typically occurs during catching for harvest and antemortem conditions at processing plants (Sparrey & Kettlewell, 1994). Anecdotal evidence indicates that the struggling behavior of shackled broiler chickens may be negatively linked to welfare and meat quality (Satterlee et al., 2000). One of the biggest contributions to struggling behavior is live-bird hanging caused discomfort during the antemortem period, and thereby negatively affecting the welfare of the birds (Satterlee et al., 2000). Numerous factors present in the commercial processing plants (e.g., rough shackling, plant noise, unevenness and bends in the conveyor line, temporary losses of visual contact between neighboring birds, bright lights, and fear) have been qualitatively associated with the etiology of struggling behavior in shackled broiler chickens (Gregory & Bell, 1987; Sparrey & Kettlewell, 1994; Beňo et al., 2021). These stressors promote changes in the biochemical and physiological states of various cells, disrupting their functional homeostasis and promoting exhaustion of antioxidant capacity of the birds, thereby reducing the quality and quantity of meat (Salek, 2020). In addition, the struggle amplifies mortality, carcass damage, and weight loss (Prejsnar et al., 2021).

In addition to the negative effects on welfare and production, struggling behavior leads to trauma which manifests itself in a bruise. Bruises can be noted on the farm during catching and feed withdrawal and at the processing plant during unloading, hanging, and stunning. Economic analyses suggest half a billion broiler carcasses are downgraded annually due to bruises (Satterlee et al., 2000). In addition, the struggle of shackled chickens has been positively linked with the reduction in meat quality, i.e., decrease of the pH, elevation of the shear values, and an early development of rigor mortis of the muscle (Papinaho et al., 1995).

It has been proposed that reducing struggling behavior in shackled broiler chickens may associated with improving meat quality i.e., muscle tenderness, and reduction of red wing tips, bruises, and broken bones. Furthermore, limiting struggling would possibly extend the onset of rigor and alleviate its destructive physiological effects (i.e., weaken glycolysis, decreased muscle ATP degradation, elevated final muscle pH, and lowered accumulation of lactic acid). These effects would, in turn, enhance muscle tenderness (Satterlee et al., 2000). Therefore, the need for attenuating the adverse physiological and behavioral consequences associated with struggling behavior of shackled broiler chickens is a great concern in the poultry industry. Targeting the gut microbiota with fecal microbiota transplantation, direct fed microbials, i.e., probiotics, has become a valuable biotherapeutic procedure for curing various diseases, including neuroinflammation-induced mental illness (Chunchai et al., 2018) and psychosocial disorders such as depression- and anxiety-like behaviors in humans and relative animal models (Smith et al., 2019) by improving the ecology of gut microflora and oxidation stability to reduce physical and physiological stress effects, and thereby to improve the economic efficiency of poultry production (Khalil et al., 2021).

Gut microbiota, as an endocrine organ (Clarke et al., 2014), functional regulating the immune system through synthesizing interferon/cytokines and immunoglobulins (IgG, IgM, and IgA), activating white blood cells like lymphocytes and natural killer cells, and boosting the activity of macrophages and heterophils (Abd El-Hack et al., 2020). In contrast, some studies have reported regulating gut microbiota without treatment effects on broiler growth performance and carcass characteristics (Salehimanes et al., 2016; Sarangi et al., 2016). Variations in the compositions or concentrations of the probiotics may be responsible for those conflicting findings. Furthermore, the effect of the probiotic, *Bacillus subtilis*, on struggling behavior and related meat quality in shackled broiler chickens has not been examined, although it, as a probiotic, has been widely used in farm animals including broilers. In broilers, most of the *Bacillus subtilis* investigations have been concentrated on physiological changes and production performance under regular production conditions (Goodarzi Boroojeni et al., 2018) and various stressful conditions, such as heat stress (Wang et al., 2018) and *Salmonella* challenge (Abudabos et al., 2019).

Therefore, the objective of this study was to investigate the effects of the probiotic, *Bacillus subtilis,* on the struggling behavior and related meat quality and immune response of shackled broiler chickens. We hypothesized that the dietary probiotic supplement would mitigate preslaughter stress reactions to improve immune response and meat quality in broilers.

## MATERIALS AND METHODS

All procedures and bird handling were approved by the Animal Care and Use Committee of the Faculty of Veterinary Medicine, Assiut University, Egypt (Number: 06/2023/0068).

### Probiotic

A Probiotic (CLOSTAT HC SP Dry, Kemin, Europe, NV; Herentals, Belgium) was utilized in this study. CLOSTAT contained a unique, patented spore-forming strain of *Bacillus subtilis* PB6 which was isolated from chickens who had survived postexposed to *Clostridium perfringens* under a regular rearing environment. CLOSTAT was developed for reducing intestinal pathogenic colonization which is one of the major issues causing sickness and death in poultry (Teo & Tan, 2007). CLOSTAT has been widely used in poultry production for increasing bird health and welfare (Mohammed et al., 2021)

### Birds and Housing

Two hundred and ten 1-day-old male broiler chicks (Ross 708 strain; El-wade, Assiut, Egypt) were weighed and allocated to 21 floor pens (10 birds per 100 cm × 100 cm floor pen) with similar average body weight in an environment-controlled room (The Animal and Poultry Behavior and Management Research Unit, Assiut University, Egypt). Fresh and dry wood shaving as bedding was used at a depth of 5 cm. The bird management was according to the guidelines of (Aviagen, 2018). Environmental temperature in the first week of bird life was 35°C and gradually decreased to 26°C (0.5°C/d.) till the end of experiment. Relative humidity inside the pen was about 50%. An intermittent light program (10 lux) was applied, starting from 23 h light:1 h dark (the first 7 d of age), then gradually decreased to 20 h light: 4 h dark till the end of experiment.

### Experimental Design

The 21 floor pens were randomly allocated to 1 of 3 dietary treatments in 7 replicates of 10 broilers per replicate: a regular diet mixed with the probiotic, *Bacillus subtilis* PB6, at 0 (control), 0.25 (0.25×), and 0.5 (0.5×) g/kg feed (n = 7). The concentrations of CLOSTAT dietary treatments were based on the company's recommendation. The dietary treatment was from d 1 to d 35 when they reached the market weight (Table 1). A small amount of the basal diet was first mixed with the respective amounts of probiotic as a small batch, and then with a larger amount of the basal diet until the total amount of each respective diet was homogeneously mixed. The birds were fed with starter diet from d 1 to 14, followed by grower diet from 15 to 28 d of age, and then a finisher diet from 29 to 35 d. Water was provided in clean drinkers at all pens (Mohammed et al., 2019). The birds were harvested at d 35. Behavior observation was conducted, and blood and leg and breast muscles were collected for assessing the treatment effects on struggling behavior, serum concentrations of immunoglobulins, meat antioxidant capacity, meat quality, and sensorial tasty.

**Table 1:** Components of base diet.^1^

Ingredient %	Starter (1–14d)	Grower (15–28d)	Finisher (29–35d)
Corn ground	57.66	63.76	66.9
Soybean meal 47.5%	35.27	29.68	26.3
Soybean oil degummed	3	3	3.52
Calcium carbonate	1.41	1.38	1.49
Phosphate moncocalcium	1.42	1.02	0.82
L-Lysine	0.11	0.1	0.02
Salt plain	0.48	0.46	0.48
L-Threonine 98%	0.06	0.04	0
DL-Methionine	0.24	0.21	0.12
Poultry turkey starter	0.35	0.35	0.35
Calculated analysis			
Crude protein %	23.4	22.8	19.2
Poultry ME kcal/kg	3050	3151	3200
Calcium %	0.95	0.85	0.75
Available phosphorus %	0.50	0.44	0.36
Methionine %	0.66	0.59	0.53
Methionine+Cystine %	1.04	0.97	0.86
Lysine %	1.42	1.29	1.09
Threonine %	0.97	0.89	0.74
Na %	0.22	0.20	0.19

Provided per kilogram of diet: vitamin A, 13.233 IU; vitamin D3, 6.636 IU; vitamin E, 44.1 IU; vitamin K, 4.5 mg; thiamine, 2.21 mg; riboflavin, 6.6 mg; pantothenic acid, 24.3 mg; niacin, 88.2 mg; pyridoxine, 3.31 mg; folic acid, 1.10 mg; biotin, 0.33 mg; vitamin B12, 24.8 μg; choline, 669.8 mg; iron from ferrous sulfate, 50.1 mg; copper from copper sulfate, 7.7 mg; manganese from manganese oxide, 125.1 mg; zinc from zinc oxide, 125.1 mg; iodine from ethylene diamine dihydroidide, 2.10 mg; selenium from sodium selenite, 0.30 mg.

1 The ration formulation was produced according to Aviagen (2018). A regular diet supplemented with 0 (Control) 0.25 (0.25X) and 0.5 (0.5X) g kg^−1^probiotic.

### Struggling Behavior Test

At 35 day-old, the birds were subjected to feed withdrawal at 08:00 AM and thereafter 105 birds (35 birds per treatment) were crated, then transported in an open bed of a pick-up truck for a journey of 80 km (90 min approximately) to the automatic poultry abattoir (Industrial Safa City, Bani Ghaleb, Assiut, Egypt). Each crate contained 5 pen mates, and then 2 randomly tested birds per pen (crate) (14 birds per treatment) were identified via a small spray paint mark on the backs of their necks. Behavioral observation as well as physiological measurements began after the birds had arrived at the abattoir. At test, broilers were carried in an upright position by 2 trained experimenters to the test room and were hanged by their legs from adjacent shackles of a moving processing line. The leg apertures were fixed at 1.1 cm along the whole 18 cm length of the shackle (Satterlee et al., 2000).

Upon removal of the shackler hands from the tested birds, 1 min observation period was used to record struggling behaviors: the number of vocalizations, the latency until the first struggle (in seconds), the numbers of struggling bouts, and the accumulated time spent struggling (in seconds) were recorded.

### Meat and blood Sample collection

According to the traditional Islamic Halal Method (Shahdan et al., 2016), the selected birds (used for struggling behavior observation) (2 birds per pen) were sacrificed by cutting through their jugular veins for blood collection, then bleeding for 120 s for carcass collection. The collected blood samples were kept at room temperature for 1 h, and then centrifuged at 3000 × g for 15 min at 4°C. The serum samples were stored at −20˚C until analyzing the levels of bird's immune response.

The carcasses were semi-scalded at 54°C for 30 s before being manually plucked. Followed evisceration and washing, the carcasses were drained for 10 mins. After draining, the right side of each bird was separated as leg (thigh), and breast muscle, and kept at 3°C ± 0.5°C for 30 min and 5 h, respectively, in accordance with the conventional farm fresh meat technique (Mohammed et al., 2021) for meat quality evaluation.

### Immune Response

Serum concentrations of immunoglobulins (IgG, IgM, and IgA) were detected using the commercial poultry enzyme-linked immunosorbent assay kits followed by the manufacturer's instruction (FAR; Fermi, Verona, Italy).

### Meat Antioxidant Capacity Analysis

#### Total Antioxidant Capacity

The Total antioxidant capacity (**TAC**) of the breast muscles was calculated using the commercial kits (Egyptian Company of Biotechnology, Cairo, Egypt) with a digital spectrophotometer (Cecil instrument, Cambridge, England) by following the manufacturer's instructions.

#### Lipid Peroxidation Analysis

Malondialdehyde (**MDA**) concentration of the breast muscles was calculated using an MDA commercial kit (Egyptian Company of Biotechnology, Cairo, Egypt) with a digital spectrophotometer (Cecil instrument, Cambridge, England) by following the manufacturer's instructions.

#### Protein Oxidation

The measure was done by using a protein carbonyl commercial kit (Egyptian Company of Biotechnology, Cairo, Egypt) with a digital spectrophotometer (Cecil instrument, Cambridge, England) following the manufacturer's instructions and protein oxidation of the breast muscles was estimated as protein carbonyls.

### Meat Quality Evaluation

#### pH Value

The pH values of the cooled leg and breast muscles were measured using a calibrated HI-99163 Food Care pH Meter (Hanna Instruments) at 3 distinct places at 30 min and 5 h postslaughter (Mohammed et al., 2021). Leg and breast meat pH values were analyzed and its mean was reported.

#### Color Level

Using the CIE Lab Color System (1976), which includes CIE L* (lightness), a* (redness), and b* (yellowness), the color values of the leg and breast meat from the 5-h postslaughter group were calculated (Mohammed et al., 2021). A CR-400 Chroma Meter was used to take 2 random readings on each leg and breast muscle. For statistical analysis, the mean was computed for each sample of breast and leg tissue, separately, according to the American Meat Science Association's recommendations for measuring color (AMSA, 2012).

#### Water-Holding Capacity

The meat from the 5 h group was used to determine the meat's water-holding capacity (**WHC**%). The estimate WHC% was created using the percentage of meat weight loss that occurs when pressure is applied to the muscle (Mohamed et al., 2021). Concisely, 0.5 g of the meat cubes from the same part of each sampled leg and breast muscle was placed as a sandwich between 2 filter papers and 2 glass plates, and then a 10-kg weight was placed on the top glass plate for 5 min. The difference between the muscle weight before and after weight loading was used to quantify the water loss. The percentage of exudate water in respect to the initial sample weight was used to represent the results.

#### Cooking Loss

An oven that had been preheated to 170^◦^C was used to measure the meat cooking loss (**CL**) of the meat samples collected from the 5 h postslaughter group using a previously described method (Mohammed et al., 2021). Concisely, the unprocessed breast and leg muscle samples were weighed, placed in stainless steel trays with parchment paper, and dried for 30 min in an incubator (Thomas Scientific SMI2), then placed inside the oven and until the internal temperature reached 75°C. A data logger was used to keep track of the temperature of the meat. The percentage difference between the samples' initial and final weights was used to calculate the CL.

### Sensory Analysis

In a nutshell, the sensory test was run 24 h after the slaughter. After pretreated with 1% (w/w) salt, the meat samples were roasted in a preheated oven (190^°^C) until the internal temperature reached 75°C. For testing, a sensory crew examined the samples after standardizing their size, shape, and temperature. The following factors were evaluated using a 9-point hedonic scale: flavor (the sensation of smell and taste released from the samples before and during chewing), texture (perception of the strength required to shear the samples when biting), preference (the sum of all sensory perceptions, expressing the sensory team's assessment of the quality of the product), and general aspect (ideation of the product). The test team provided the median values for each sensory component were used for analysis (Mohammed et al., 2021).

### Statistical Analysis

The experimental design was performed in a completely randomized design. The overall effect of the probiotic supplementation on broiler chickens struggling behavior, immune response and meat quality was analyzed by 1-way ANOVA, with the pen was the experimental unit (n = 7). Means of the data were analyzed by using PROC MIXED model with SAS 9.4 software (SAS Institute Inc., Cary, NC). The probiotic treatment was the fixed effect, and the 2 birds within a pen served as a subsample. The normality of the data was analyzed by the Shapiro–Wilk test. Individual differences between groups were tested by the Tukey–Kramer test when a significant main effect was detected. Least square means and SE were presented, and statistical significance was set at *P* < 0.05 and *P* < 0.01.

## RESULTS

### Struggling Behavior Test

The probiotic effect on the struggling behavior is presented in Table 2. The struggling behavior test parameters (number of vocalizations, the latency until the first struggle, the numbers of struggling bouts, and the accumulated time spent struggling) were not affected by the probiotic supplementation although control birds had a longer accumulated struggling time (5.33 sec vs. 2.26 sec) and shorter latency to the first struggle (7.16 Sec vs. 25,00 Sec) than 0.5× birds (*P* > 0.05).

**Table 2:** Effect of dietary supplementation of different levels of a probiotic (*Bacillus subtilis*) on the struggling behavior of shackled broiler chickens exposed to preslaughter stress.

1Treatment	Control	0.25×	0.5×	*P*-value
Number of vocalizations	1.50 ± 0.56	1.83 ± 0.47	1.00 ± 0.45	0.5079
Numbers of struggling bouts	1.50 ± 0.43	1.50 ± 0.22	1.33 ± 0.33	0.9229
Accumulated time spent struggling (sec)	5.33 ± 2.02	4.66 ± 1.14	2.16 ± 0.60	0.2666
Latency the first struggle (sec)	7.16 ± 4.71	18.00 ± 3.96	25.00 ± 8.95	0.1633

1 The data were collected from 14 birds per treatment (2 birds per pen x 7 pens per treatment) and presented as Mean + SE (n = 7).

### Immune Response

The effect of the probiotic on the serum IgG, IgM, and IgA is presented in Table 3. Compared to controls, the 0.25X group had lower levels of serum IgM (10.40 mg vs. 6.60 mg; *P* < 0.05), but the difference disappeared between the 0.25× and 0.5× groups (6.60 mg vs. 7.18 mg; *P* > 0.05). There was no effect of the probiotic regardless of dose on the levels of serum IgG and IgA in shackled broilers exposed to preslaughter stress (*P* > 0.05).

**Table 3:** Effect of dietary supplementation of different levels of a probiotic (*Bacillus subtilis*) on serum concentration of IgG, IgA, and IgM of the shackled broiler chickens exposed to preslaughter stress.

1Treatment	Control	0.25×	0.5×	*P*-value
IgG (mg/dL)	153.00 ± 13.56	146.60 ± 12.86	148.00 ± 8.38	0.9441
IgA (mg/dL)	40.17 ± 0.67	47.40 ± 2.88	41.17 ± 1.12	0.1570
IgM (mg/dL)	10.40 ± 0.71^a^	6.60 ± 0.47^b^	7.180 ± 0.55^ab^	0.0231

^a-b^Least square means with different superscripts in the same row differ significantly (Mean ± SE. *P* < 0.05, n = 7).

1 The data were collected from 14 birds per treatment (2 birds per pen x 7 pens per treatment).

### Meat Quality

#### Meat Antioxidant Capacity Analysis

The effect of the probiotic on the breast meat antioxidant capacity (TAC, MDA, and protein carbonyls) is presented in Table 4. Compared to controls, both 0.25× and 0.5× probiotic fed broilers had higher breast muscle concentration of TAC (0.34 mM/L vs. 1.20 mM/I vs. 1.34 mM/L, control vs. 0.25× vs. 0.5×; *P* < 0.05) but lower protein carbonyls (7.07 mmol/mL vs. 5.53 mmol/mL vs. 5.39 mmol/mL, control vs. 0.25× vs. 0.5×; *P* < 0.05). There was no effect of the probiotic regardless of dose on the breast muscle concentration of MDA (*P* > 0.05).

**Table 4:** Effect of dietary supplementation of different levels of a probiotic (*Bacillus subtilis*) on antioxidant activities of breast muscle of the shackled broiler chickens exposed to preslaughter stress.

1Treatment	Control	0.25×	0.5×	*P*-value
Protein carbonyls (mmol/mL)	7.07 ± 0.47^a^	5.53 ± 0.11^b^	5.39 ± 0.15^b^	0.0052
Malondialdehyde (nmol/g)	11.38 ± 0.36	11.41 ± 0.37	11.57 ± 0.13	0.8059
Total antioxidant activity (mM/L)	0.34 ± 0.08^b^	1.20 ± 0.20^a^	1.34 ± 0.20^a^	0.0056
				

^a-b^Least square means with different superscripts in the same row differ significantly (Mean ± SE. *P* < 0.05, n = 7).

1 The data were collected from 14 birds per treatment (2 birds per pen x 7 pens per treatment).

#### Meat General Sensory Analysis

The effect of the probiotic on the outcomes of general sensory analysis and preference of leg and breast muscles of shackled broilers exposed to preslaughter stress are presented in Tables 5. There was a treatment effect on meat taste. Compared with controls, the leg and breast meat from probiotic fed broilers had better outcomes in the general sensory analysis (*P* < 0.01). The measured general sensory parameters of the leg and breast meat were in the order: control vs. 0.25× vs. 0.5×; flavor, 5.30 vs. 7.90 vs. 8,50; texture, 5.40 vs. 7.50 vs. 8.20; preference, 5.10 vs. 7.80 vs. 8.30; and general aspect. 5.80 vs. 7.60 vs. 8.00.

**Table 5:** Effect of dietary supplementation of probiotic (*Bacillus subtilis*) on the general sensory of leg and breast meat of shackled broiler chickens exposed to preslaughter stress.

Kind of meat	Leg meat				Breast meat			
1Treatment	Control	0.25X	0.5X	*P*-value	Control	0.25×	0.5×	*P*-value
Flavor	5.30 ± 0.25^b^	7.90 ± 0.25^a^	8.50 ± 0.25^a^	0.0001	5.30 ± 0.25^b^	7.90 ± 0.25^a^	8.50 ± 0.25^a^	0.0001
Texture	5.40 ± 0.22^b^	7.50 ± 0.22^a^	8.20 ± 0.22^a^	0.0001	5.40 ± 0.22^b^	7.50 ± 0.22^a^	8.20 ± 0.22^a^	0.0001
Preference	5.10 ± 0.17^b^	7.80 ± 0.17^a^	8.30 ± 0.17^a^	0.0001	5.10 ± 0.17^b^	7.80 ± 0.17^a^	8.30 ± 0.17^a^	0.0001
General aspect	5.80 ± 0.36^b^	7.60 ± 0.36^a^	8.00 ± 0.36^a^	0.0001	5.80 ± 0.36^b^	7.60 ± 0.36^a^	8.00 ± 0.36^a^	0.0001

^a-b^Least square means with different superscripts in the same row differ significantly (Mean + SE. *P* < 0.05, n = 7).

1 The data were collected from 14 birds per treatment (2 birds per pen x 7 pens per treatment).

#### Meat pH, Color, Water-holding Capacity (WHC%), and Cooking Loss (CL)

The effect of the probiotic on leg and breast meat pH, color, WHC%, and CL of shackled broiler chickens exposed to preslaughter stress is presented in Tables 6. The pH values were reduced in the leg and breast meat from the probiotic-fed shackled broilers at 30 min and was further reduced at 5 h after slaughter than those of controls (*P* < 0.01). Furthermore, compared with controls at 5 h after slaughter, the lightness, redness, and yellowness of leg and breast meat were significantly higher in the probiotic-supplemented shackled birds (*P* < 0.01). Water-holding capacity of leg and breast meat was increased in the probiotic-fed shackled birds compared with controls (from 68.80%, controls up to 91.00%, 0.5× birds in the leg meat and 70.40%, control up to 93.20%, 0.5× birds in the breast meat; *P* < 0.01); consequently, the leg meat CL was reduced in the probiotic-fed groups (*P* < 0.01) although the breast meat CL was reduced only in the 0.5× broilers (controls vs. 0.5× birds: 43.06 vs. 24.07 of the leg meat and 30.31 vs. 16.46 of the breast meat; *P* < 0.01) but not in the 0.25× broilers compared to controls (*P* > 0.05).

**Table 6:** Effect of dietary supplementation of probiotic (*Bacillus subtilis*) on the pH, color, water-holding capacity (WHC%), and cooking loss (CL) of leg and breast meat of shackled broiler chickens exposed to preslaughter stress.

Kind of meat	Leg meat				Breast meat			
1Treatment	Control	0.25×	0.5×	*P*-value	Control	0.25×	0.5×	*P*-value
pH								
30 min after slaughter	6.49 ± 0.04^a^	6.16 ± 0.04^b^	6.14 ± 0.04^b^	0.0001	6.46 ± 0.03^a^	6.04 ± 0.03 ^b^	6.12 ± 0.03^b^	0.0001
5 h after slaughter	6.12 ± 0.06^a^	5.64 ± 0.06^b^	5.30 ± 0.06^c^	0.0001	6.08 ± 0.05 ^a^	5.58 ± 0.05 ^b^	5.20 ± 0.05^c^	0.0001
Color								
Lightness (L∗)	51.06 ± 0.97^b^	56.02 ± 0.97^a^	58.28 ± 0.97^a^	0.0035	51.06 ± 0.97^b^	56.02 ± 0.97^a^	58.28 ± 0.97^a^	0.0007
Redness (a∗)	18.43 ± 0.41^b^	20.68 ± 0.41^a^	21.84 ± 0.41^a^	0.0007	18.43 ± 0.41^b^	20.68 ± 0.41^a^	21.84 ± 0.41^a^	0.0003
Yellowness (b∗)	7.62 ± 0.13^b^	8.24 ± 0.13^a^	8.54 ± 0.13^a^	0.0009	7.62 ± 0.13 ^b^	8.24 ± 0.13^a^	8.54 ± 0.13^a^	0.0008
WHC%	68.80 ± 1.55^c^	82.20 ± 1.55^b^	91.00 ± 1.55^a^	0.0001	70.40 ± 1.42^c^	88.40 ± 1.42^a^	93.20 ± 1.42^a^	0.0001
CL	43.06 ± 0.27^a^	34.36 ± 2.27^b^	24.07 ± 2.27^c^	0.0003	30.51 ± 3.40^a^	23.18 ± 3.40^ab^	16.46 ± 3.40^b^	0.0001

^a-b^Least square means with different superscripts in the same row differ significantly (Mean + SE. *P* < 0.05, n = 7).

1 The data were collected from 14 birds per treatment (2 birds per pen x 7 pens per treatment).

## DISCUSSION

In a processing plant, large number of broiler chickens show wing-flapping and violent struggling when they are inverted, hanged upside down by clamped their legs into stirrups during live-shackles slaughter, and some birds even climb the shackles (Sparrey & Kettlewell, 1994; Kannan et al., 1997). Struggle of shackled birds has a profound deleterious impact on broiler meat quality and well-being. It also suppresses immunity and disrupts physiological homeostasis, leading to hypersensitivity causing discomfort and bruises; consequently, leading to carcass downgrading (Anonymous, 1997). These reactions have a harmful effect on meat quality through an early lowering of muscle pH and increasing speed of rigor mortis and muscle shear values (Ma & Addis, 1973). Violent struggling and wing flapping responses at the point of shackling have also been anecdotally associated with compression of the birds’ hocks due to tightfitting shackles (Gregory & Bell, 1987) as well as wing broken (Knierim & Gocke, 2003; Cockram et al., 2020).

Probiotic supplementation has been used as a growth promotor for increasing body weight and bone mass density (Lan et al., 2016; Mohammed et al., 2021) and used as a biotherapy for reducing a variety of diseases, including neuroinflammation via the gut-brain axis (Zhu et al., 2020). These outcomes indicate that microbes in the gut, as immune and neuronal modulators, have a significant effect on brain health (Malmir et al., 2021; Ansari et al., 2023). In the present study, the struggling behavior was not affected by the probiotic supplementation regardless of its level. Failure to observe any treatment effects could be associated with multiple factors, such as chicks’ age, the length of stimulation, the type of probiotic and its concentration as well as the length of feeding time when the test was conducted. The current results indicate that the probiotic under the current condition may be not suitable for suppressing acute stress induced pain and uncomforted sensation via the brain cognitive, emotional processing center, the Anterior Cingulate Cortex (**ACC** plus insula) (De Ridder et al., 2021).

In broiler chickens, exposure to stress, especially chronic stress, causes immunosuppression and endocrine disturbance (Lee et al., 2016; Mohammed et al., 2019), ultimately resulting in reduction in production performance. Probiotics could affect the Peyer's patches cells in the ileum and help in boosting their mucosal immune system by production of IgA antibodies (Corthésy et al., 2007). However, the concentrations of immunoglobulins (Ig A, IgM, and IgG) in the intestine and blood are affected by various stimulations. In the current study, the serum levels of IgM were reduced in the 0.25× group compared to controls following preslaughter stress. Similar to the findings, Deng et al. (2012) reported that dietary inclusion of the probiotic contained *Bacillus licheniformis* reduced the immunoglobulins levels in the cecum and ileum of laying hens exposed to heat stress. However, present and previous studies evidenced that probiotic treatments didn't cause a difference in the levels of IgG and IgA antibodies in broiler chickens (Mahfuz et al., 2017; El-Hack et al., 2020). Reduction of the serum levels of the IgM in the 0.25× birds suggests the role of the probiotic in lowering excessive proinflammatory cytokines to maintain a normal cytokine environment by restoring the integrity of the gut epithelium after stress (Deng et al., 2012).

Exposure of birds to stress may result in pathophysiological changes which potentially lead to deterioration in meat quality, including reduction in protein synthesis and turnover and increase in ante/postmortem glycolytic metabolism, causing excessive formation of reactive oxygen species (**ROS**). Normally, ROS, as a group of stress sensors, play key roles contributing to the defense mechanisms to maintain biological homeostasis. Stress causes overproduction of ROS leading to pathophysiological changes not only to internal (or vital and metabolic) organs but also to skeletal tissues. Consequently, it leads to negative effects on protein functionality and oxidation stabilities of chicken skeletal muscles (Azad et al., 2010). Recent study noted that dietary supplementation of the probiotic composed of *Bacillus subtilis* in broiler chicken diets alleviates oxidative damage in the breast muscle after exposure to stress (Cramer et al., 2018). In the present study, dietary supplementation of the probiotic, *Bacillus subtilis,* reduced the protein oxidation (estimated as protein carbonyls) but increased the TAC of breast muscle of broiler chickens exposed to preslaughter stress. The improvement in the protein oxidation and TAC could be attributed to the improvement of the chaperon proteins such as heat shock proteins as reported previously (Ghosh et al., 2018). These proteins support the recovery of cell membranes by promoting the folding and denaturing the freshly synthesized proteins to preserve cell homeostasis (Zhang et al., 2015).

The general aspect, preference, texture, and flavor are the most important and perceptible meat features, which influences the initial and final meat quality judgment by consumers before and after purchasing a meat product (Cross et al., 1986). In the current study, dietary supplementation of probiotic, *Bacillus subtilis*, improved the sensory properties of the leg and breast muscles of shackled broiler chickens exposed to preslaughter stress. Khan et al. (2018) also noted an improvement in the meat quality and the sensory properties of leg meat of broiler chickens supplemented with a probiotic containing *Lactobacillus acidophilus* and *Streptococcus cerevisiae*. However, Stęczny and Kokoszynski (2019) reported no effect of dietary supplementation of a probiotic contained *Lactobacillus spp*., *Bifidobacterium spp*., *Lactococcus spp*., *Streptococcus thermophilus, Bacillus subtilis, Rhodopseudomonas spp*. and *Saccharomyces cerevisiae* yeast on the sensory properties of breast muscle of broiler chickens. In the current study, the probiotic improving the sensory properties of breast and leg muscle of shackled broiler chickens could be attributed to the improvement in the lipid metabolism through preventing the meat storage-induced oxidative stress (Bai et al., 2016).

It has been well documented that stress can reduce protein contents of chicken breast muscles with undesirable meat quality as indicated by the changes in the color, WHC%, tenderness, and oxidative stability (Kim et al., 2017). Meat quality is determined by several variables; however, the pH, WHC%, and tenderness are particularly significant effectors. Short-term preslaughter associated stressors, like fasting, transport, and shackling, can influence these features. Metabolic fatigue can also be caused by rough handling, congestion, heat stress, struggling as well as vehicle vibration during harvest related shackling and transportation discomfort. Preslaughter stress also contributes to the formation of body injury, bone fracture and tissue bruises; consequently, these damages caused capillary rupture may lead to ecchymosis, hemorrhage, and or meat splash. All those changes can modify muscle metabolism, leading to unfavorable alterations in meat quality (Castellini et al., 2016). Modulation of diets has become an alternative managerial strategy for improvement of meat quality in broilers (Tavaniello et al., 2018; Mohammed et al., 2021; Sampath et al., 2021). In the current study as well as previously published studies, it shows that dietary supplementation of probiotic, *Bacillus subtills*, has a promise in improving the meat attributes, such as general sensory characters, color, pH, CL, and WHC% (Zhang et al., 2015; Mohammed et al., 2021).

In the current study, the leg and breast muscle color, i.e., lightness (L∗), redness (a∗), and yellowness (b∗), were significantly increased in the probiotic-supplemented groups in comparison with controls. Similar to the findings, Khan et al. (2018) who noted an improvement in the color of the meat of heat stressed broiler chickens fed with a probiotic contained *Lactobacillus acidophilus* and *Streptococcus cerevisiae*, however, Stęczny and Kokoszynski (2019) reported no treatment effects of a probiotic contained *Lactobacillus spp*., *Bifidobacterium* spp., *Lactococcus* spp., *Streptococcus thermophilus, Bacillus subtilis, Rhodopseudomonas* spp. and *Saccharomyces cerevisiae* yeast. In the current study, the improvement in the leg and breast muscle color could be attributed to increase the muscle myoglobin contents (Han et al., 2020). Moreover, the significant impact of the probiotic supplementation in improving meat color could be linked with its ability in reducing the nitric oxide output (Ismail et al., 2019). Probiotic diets could be used as a tool for increasing economic profiles for the poultry meat industry as the consumer's acceptance of meat is significantly influenced by its color.

Meat quality is influenced by its pH levels during the biochemical reactions, rigor mortis, and conversing muscle to meat after slaughter (Sanudo, 1992). Preslaughter stress results in higher muscle pH probably due to greater glycogen depletion. During glycolysis, glucose is converted to lactic acid, which lowers the ultimate pH level. Stress has an impact on this metabolism because it causes a rise in plasma corticosterone, which in turn causes a rise in muscle glycogenolysis. Thus, by placing an acute demand on energy, stress helps to decrease glycogen stores in the liver and skeletal muscles (Castellini et al, 2016). In this study, the pH of the breast and leg muscles of shackled broiler chickens after preslaughter stress was decreased in the probiotic supplemented birds compared to controls. Khalil et al. (2021) also reported a reduction in pH from 6.29 to 6.08 in muscles of broiler chickens fed with diet containing *Enterococcus faecium*. Furthermore, Cramer et al. (2018) reported that the probiotic supplementation composed of *Bacillus subtilis* diminishes the pH values in heat stress exposed broiler muscle by likely affecting the rate of postmortem glycolysis metabolism.

Cooking loss and WHC% are another 2 important meat quality factors closely linked to its tenderness. Tenderness is among the most important functional properties of raw meat (Pelicano et al., 2003). The present findings show that dietary supplementation of the probiotic, *Bacillus subtilis*, decrease CL but increase the WHC% of leg and breast muscles of shackled broiler chickens exposed to preslaughter stress. Park and Kim (2014) also noted an improvement in the WHC% and CL of breast muscles of broiler chickens fed with probiotic, *Bacillus subtilis*. Conversely, negative effect was noted by Khalil et al. (2021) who reported a reduction in WHC% of breast muscles of broiler chickens supplemented with a probiotic contained *Enterococcus faecium*. Improvement in the WHC% of the broiler chickens muscles in this study could be linked with an improvement in the intramuscular fat content in the leg and breast muscles. The alteration in the fatty acid synthesis is contributed to meat tenderness (Yang et al., 2010).

## CONCLUSION

The present findings indicate that the *Bacillus subtilis*-based probiotic supplement improves immune and oxidative responses in shackled broiler chickens under preslaughter stress challenge. In addition, probiotic-fed broilers had improved leg and breast meat pH, color, CL, WHC%, with greater sensory properties compared with controls. The present results suggest that feeding probiotics could be a management strategy to reduce preslaughter stress-induced downgraded meat quality during poultry processing.

## DISCLOSURES

We declare no conflict of interest. The supporters had no say in the experimental design, sample collection, data analysis and interpretation and writing of the manuscript.
